# An Anesthesia-Free Pharmacological Assay for Zebrafish Heart Failure Models Using a Microfluidic Platform

**DOI:** 10.3390/biomedicines14092005

**Published:** 2026-09-06

**Authors:** Qiuyue Song, Fangrui Liu, Jinyan Zhao, Wenli Xie, Xianjun Fu, Kuo Xu

**Affiliations:** 1 Research Institute of Marine Traditional Chinese Medicine (Qingdao Academy of Chinese Medical Sciences), The SATCM’s Key Unit of Discovering and Developing New Marine TCM Drugs, Key Laboratory of Marine Traditional Chinese Medicine in Shandong Universities, Shandong University of Traditional Chinese Medicine, Qingdao 266114, China; 2 College of Pharmacy, Shandong University of Traditional Chinese Medicine, Jinan 250355, China; 3 National Center of Technology Innovation for Comprehensive Utilization of Saline-Alkali Land, Dongying 257345, China

**Keywords:** Zebrafish, heart failure, microfluidics, isoproterenol, cardiac function

## Abstract

**Background:** The zebrafish model is extensively utilized for phenotypic screening, particularly in heart failure research. However, traditional photography-based data collection requires tricaine methanesulfonate (MS-222) anesthesia, which can suppress cardiac function and introduce artifacts. This study presents a microfluidic platform designed to facilitate anesthesia-free, noninvasive in vivo photography of zebrafish heart failure models. **Methods:** Isoproterenol (ISO) induced heart failure in 1 day post-fertilization (dpf) zebrafish embryos. Larvae were assigned to three groups: Control (E3 embryo medium only), Model (1 mM ISO for 48 h from 1 dpf), and Positive (1 mM ISO + 10 μM propranolol for 48 h from 1 dpf). Subsequently, a microfluidic chip featuring tapered immobilization channels was adopted. The chip facilitated the photography and collection of cardiac function parameters from lateral and supine positions, including cardiac output (CO), blood flow velocity (BFV), ejection fraction (EF), stroke volume (SV), and fractional shortening (FS). All imaging was performed in completely independent parallel experiments, with no individual larva shared between the two imaging methods. Data obtained under hydrodynamic confinement were compared with those acquired under MS-222 anesthesia. **Results:** Cardiac parameters measured using the microfluidic platform were significantly higher than those under MS-222 anesthesia: CO (0.339 ± 0.032 vs. 0.279 ± 0.023 nL/s, *p* < 0.001), BFV (681 ± 53 vs. 604 ± 38 μm/s, *p* < 0.001), EF (10.3 ± 0.7% vs. 6.6 ± 0.7%, *p* < 0.001), SV (184,519 ± 12,822 vs. 97,979 ± 5306 μm^3^, *p* < 0.001), and FS (7.3 ± 1.8% vs. 3.7 ± 0.9%, *p* < 0.001). Strong correlations (R^2^ > 0.85, *p* < 0.0001) revealed that anesthesia underestimated parameters by 11.3–49.3%. **Conclusions:** Compared with the traditional anesthesia-based method, the microfluidic platform enables more precise cardiac parameter collection in a zebrafish heart failure model than when anesthesia is used, offering a reliable tool for phenotypic screening of zebrafish embryos.

## 1. Introduction

Cardiovascular diseases (CVDs) remain the leading cause of global mortality, accounting for approximately 18.6 million deaths annually, with heart failure (HF) representing the terminal and most debilitating stage of most CVDs [1,2,3]. Despite significant clinical advances, HF continues to impose a substantial health burden because of high hospitalization rates, poor prognosis, and limited therapeutic options [4]. The zebrafish has emerged as an indispensable vertebrate model for cardiovascular research and drug discovery because of its optical transparency during early development, high genetic tractability, and cardiac physiology that is conserved with that of humans [5,6,7,8]. Larval zebrafish develop a functional two-chambered heart within 48 h post-fertilization, and drug-induced HF models have been widely validated for studying cardiac dysfunction [9,10,11,12,13,14,15]. However, traditional in vivo photography of these models requires anesthesia to immobilize the larvae, and the gold-standard anesthetic tricaine methanesulfonate (MS-222) exerts profound dose-dependent cardiovascular depressant effects, introducing unavoidable artifacts that compromise the accuracy and physiological relevance of HF assessments [16,17,18,19,20,21,22].

Several strategies have been proposed to mitigate this limitation, including low-dose anesthesia, agarose embedding, and mechanical immobilization devices [23,24,25,26]. However, low-dose anesthesia still causes measurable cardiovascular suppression, whereas agarose embedding can restrict cardiac movement and induce physiological stress [27]. Microfluidic technology has recently shown promise for noninvasive zebrafish immobilization via hydrodynamic forces or physical confinement, but most existing platforms are designed for general photography applications and lack optimization for HF-specific cardiac function assessment [28,29]. Many require complex fabrication processes, have low throughput, or fail to maintain zebrafish in a stable physiological state for extended photography periods, leaving a critical unmet need for a robust, anesthesia-free photography platform tailored to HF research [30].

To address this gap, a simple, high-performance microfluidic platform for the anesthesia-free, noninvasive in vivo photography of zebrafish HF models was developed in this study. The platform utilizes tapered microchannels to gently immobilize larval zebrafish via hydrodynamic confinement without physical compression or pharmacological intervention [31]. We systematically evaluated its performance by comparing cardiac function parameters, photography quality, and animal viability with those of the traditional MS-222 anesthesia method. Our results demonstrate that this platform avoids anesthesia-associated artifacts, more closely reflects physiological cardiac function, and enables the accurate, reproducible assessment of drug-induced cardiac dysfunction, providing a valuable tool for advancing HF mechanism research and high-throughput cardiovascular drug discovery [22].

## 2. Materials and Methods

### 2.1. Overall Study Design

The study comprised two completely independent parallel imaging experiments. In the first experiment, larvae were imaged using the anesthesia-free microfluidic platform (Section 2.5.1); in the second experiment, an independent cohort of larvae was imaged using the conventional MS-222 anesthesia method (Section 2.5.2). No individual larva was shared between the two experiments. Within each experiment, larvae were further divided into two independent orientation-specific batches: a lateral-positioning batch for the assessment of hemodynamic parameters (BFV and CO) and an independent supine-positioning batch for the assessment of cardiac functional parameters (EF, SV, and FS). Each larva was imaged once for its assigned orientation and parameters, with three independent experiments serving as replicates (*n* = 10 larvae per biological group per experiment).

### 2.2. Experimental Animals and Reagents

Zebrafish maintenance: Transgenic strain Tg (*myl7: GFP*) zebrafish were raised and maintained under standard laboratory conditions (28.5 °C, 14 h light/10 h dark cycle). Larvae at 3 dpf were used for all experiments.

Reagents: ISO hydrochloride (Aladdin, Shanghai, China; Cat. No. D2523289) was dissolved in embryo medium to prepare stock solutions. Tricaine methanesulfonate (MS-222; Aladdin, Cat. No. F2214333) was used as the anesthetic agent. Propranolol (MedChemExpress, Shanghai, China; Cat. No. 26496) was used for the positive control group. Instruments: A stereo fluorescence microscope (M-shot, Guangzhou, China; Cat. No. MZX100) was used for supine-position imaging.

A MicroZebraLab system (ViewPoint Life Sciences, Lyon, France) was used for lateral-position imaging.

### 2.3. Microfluidic Platform Fabrication and Preparation

Zebrafish larvae were immobilized and imaged according to an established protocol [32]. Briefly, we used microfluidic chip models LS-B3a and LS-P3a, which are commercial products developed and supplied by Guangdong Longseek Testing Co., Ltd. (Guangdong, China) and can be purchased directly from the manufacturer; therefore, templates for independent production are not applicable. The chips contained tapered trapping channels (inlet: 2.1 mm; outlet: 100 μm; height: 0.5 mm; length: 9.0 mm), with height restricted to 150 μm for lateral positioning (LS-B3a) and width restricted to 150 μm for supine positioning (LS-P3a) [32]. Two distinct microfluidic chips were utilized to hydrodynamically trap unanesthetized zebrafish larvae within microchannels, enabling larval orientation in either a supine or a lateral posture. The chip was then placed on the stage of the corresponding imaging device: the lateral-specific chip (LS-B3a) on the ViewPoint Heart Beat and Blood Flow System, and the supine-oriented chip (LS-P3a) under the stereo fluorescence microscope (M-shot, MZX100). These two distinct physical chips were used for two separate, independent imaging batches of larvae; they were not used sequentially on the same larva, and there is no single integrated chip accommodating both orientations.

Preoperation preparation: The microfluidic chip was primed with embryo medium containing E3 to prevent nonspecific adhesion. A syringe pump (model: MBD-1; Guangdong Longseek Testing Co., Ltd., Guangzhou, China ) was used to control the flow rate at 20 mL/h.

### 2.4. ISO-Induced Zebrafish Heart Failure Model

Larval zebrafish at 1 dpf were randomly divided into three groups: the Control group (cultured in E3 embryo medium only), the Model group (treated with 1 mM ISO for 48 h from 1 dpf), and the Positive group (treated with 1 mM ISO + 10 μM propranolol for 48 h from 1 dpf). The ISO concentration (1 mM) and exposure duration (48 h) were selected based on previously established protocols that reliably induce heart failure phenotypes in larval zebrafish [14,33].

All treatments were performed in 6-well plates with 10 larvae per well maintained at 28.5 °C under standard light conditions.

After treatment, the larvae were washed three times with fresh embryo medium before the experiments were performed.

### 2.5. Experimental Design for Dual-Position Photography

#### 2.5.1. Anesthesia-Free Photography Using the Microfluidic Platform

Individual zebrafish larvae were loaded into the microfluidic chip inlet and transported to the tapered channels by hydrodynamic flow.

- Lateral position measurement: A lateral-specific microfluidic chip (model: LS-B3a; Guangdong Longseek Testing Co., Ltd., Guangzhou, China) was used, and larvae were imaged on the ViewPoint Heart Beat and Blood Flow System (model: MicroZebraLab; ViewPoint Life Sciences Inc., Lyon, France), with 15-s continuous videos recorded. Subsequent offline image analysis was performed to calculate the CO and BFV.
- Supine position measurement: An independent supine-oriented microfluidic chip (LS-P3a) was used to fix larvae in the dorsal posture to visualize ventricular contraction under the stereo fluorescence microscope (model: MZX100; M-shot; Guangzhou Micro-shot Technology Co., Ltd., Guangzhou, China). One-second videos were captured at 20 fps for the post-processing quantification of EF, SV, and FS.

After being photographed, the larvae were recovered from the chip and maintained in embryo medium for viability assessment.

#### 2.5.2. Traditional Anesthesia Photography (Control Method)

Larvae from an independent cohort, separate from those used for microfluidic photography, were anesthetized with 300 μM MS-222 in E3 embryo medium and then mounted on glass slides with 2% methylcellulose [34]. Identical dual-position photography protocols (lateral and supine) were used as described in Section 2.5.1 to measure the same five cardiac parameters. Photography was carried out promptly after anesthesia to ensure the stable physiological status of the larvae.

### 2.6. Image Analysis and Data Quantification

Cardiac function parameters were analyzed using ImageJ-Pro Plus software (version 6.0; Media Cybernetics, Inc., Rockville, MD, USA) and the Zebrafish Blood Flow Analyzer [35].

#### 2.6.1. Lateral Position Parameters

- BFV: Calculated by tracking the movement of red blood cells in the dorsal aorta (DA) over consecutive frames.
- CO: Calculated using the flow area method, CO = V × A, where V is the average blood flow velocity in the DA and A is the cross-sectional area of the DA derived from the vessel diameter (D). Specifically, A was calculated as A = π (D/2)^2^, where D is the inner diameter of the DA measured perpendicular to the vessel axis on bright-field images acquired at the same focal plane used for BFV tracking.

#### 2.6.2. Supine Position Parameters

- Ventricular volumes were determined from the supine-position image sequences as follows. End-diastolic (ED) and end-systolic (ES) frames were identified as the frames showing the ventricle with maximum and minimum areas, respectively. The ventricular endocardial border was manually traced using the polygon selection tool in ImageJ, which fitted the contour to an ellipse and provided the major (L) and minor (D) axis lengths in μm. EDV and ESV were then calculated using the ellipsoid formula, V = (π/6) × L × D^2^, with L and D measured at end-diastole and end-systole, respectively.
- SV = EDV − ESV (μm^3^).
- EF = (SV/EDV) × 100%.
- FS = ((D_ED − D_ES)/D_ED) × 100%, D = minor axis.

### 2.7. Statistical Analysis

All data are presented as the mean ± SD from three independent experiments (*n* = 10 larvae per biological group per experiment). The statistical unit was the individual larva within each orientation-specific cohort: cardiac functional parameters (EF, SV, and FS) were analyzed in the independent supine cohort, and hemodynamic parameters (BFV and CO) were analyzed in the independent lateral cohort; within each cohort, each larva was imaged once and contributed one independent measurement. This sample size was chosen in accordance with established practices in zebrafish cardiac research [14,33].

Statistical comparisons between the microfluidic method and the anesthesia method were performed using unpaired Student’s *t*-tests, and comparisons among the three groups (Control, Model, and Positive) were performed using one-way ANOVA followed by Tukey’s post-hoc test. Correlation analysis between different cardiac parameters was conducted using Pearson’s correlation coefficient. A *p* value < 0.05 was considered to indicate statistical significance. All statistical analyses were performed using GraphPad Prism software (version 9.5; GraphPad Software, San Diego, CA, USA).

## 3. Results

### 3.1. Assessment of Cardiac Function in ISO-Induced HF Models Using the Traditional Anesthesia Method

To establish the heart failure model, zebrafish larvae at 1 day post-fertilization (dpf) were treated with 1 mM isoproterenol (ISO) for 48 h, following previously reported protocols [14,33]. Larvae were assigned to three groups: the Control group (E3 embryo medium only), the Model group (1 mM ISO for 48 h from 1 dpf), and the Positive group (1 mM ISO + 10 μM propranolol for 48 h from 1 dpf). The two imaging methods (microfluidic chip imaging and MS-222 anesthesia imaging) were applied to independent parallel cohorts of larvae, enabling a parallel comparison between the two imaging modalities rather than a sequential within-subject comparison.

Anesthesia-related outcomes: After anesthesia induction with 300 μM MS-222, no mortality was observed among the HF-stage larvae during the 1 h photography session. All the larvae were immobilized within 5 min of anesthesia exposure.

Cardiac parameters measured under anesthesia:

- Lateral position: The cardiac output (CO) was 0.279 ± 0.023 nL/s, and the blood flow velocity (BFV) was 604 ± 38 μm/s.
- Supine position: The ejection fraction (EF) was 6.6 ± 0.7%, the stroke volume (SV) was 97,979 ± 5306 μm^3^, and the fractional shortening (FS) was 3.7 ± 0.9%.

### 3.2. Assessment of Cardiac Function in ISO-Induced HF Models Using the Anesthesia-Free Microfluidic Platform

The developed microfluidic platform with tapered hydrodynamic confinement channels enabled efficient and noninvasive immobilization of ISO-treated larval zebrafish.

Platform performance in the HF models: The overall immobilization success rate was >95%, with >90% of the larvae correctly oriented for both lateral and supine position photography without physical compression or cardiac movement restriction. After 1 h of continuous dual-position photography, all larvae were successfully released from the chip and remained alive upon visual inspection under a stereomicroscope (*n* = 10 per experiment, three independent experiments). No procedure-related mortality or morphological abnormalities were observed during the 24 h post-recovery observation period.

Cardiac parameters measured by microfluidics:

- Lateral position: The CO was 0.339 ± 0.032 nL/s, and the BFV was 681 ± 53 μm/s.
- Supine position: The EF was 10.3 ± 0.7%, the SV was 184,519 ± 12,822 μm^3^, and the FS was 7.3 ± 1.8%.

**Figure 1:**
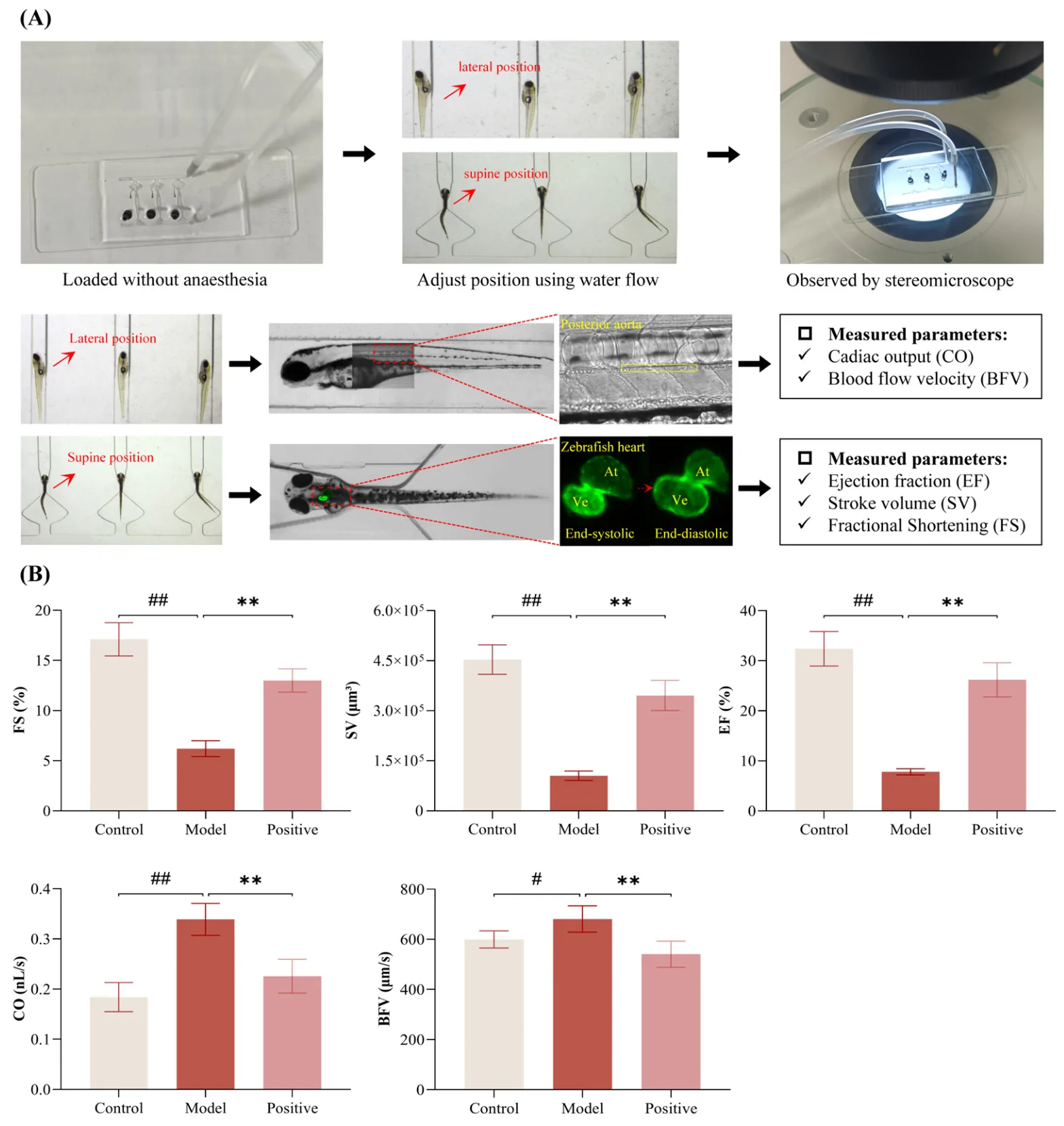
Microfluidic platform for anesthesia-free immobilization and representative cardiac images. (**A**) Schematic workflow of tapered microfluidic chip loading, posture adjustment and dual-mode cardiac parameter testing. (**B**) Quantification of cardiac function parameters (see Table S2). ^##^ *p* < 0.01, ^#^ *p* < 0.05 vs. the control group; ** *p* < 0.01 vs. the model group.

Pathological phenotype visualization: The platform allowed clear visualization of ISO-induced HF-specific pathological changes, including ventricular dilation, reduced wall thickening, and abnormal blood flow patterns (Figure 1).

### 3.3. Correlation Analysis Between the Two Methods

Quantitative comparisons and correlation analyses were performed to evaluate the impact of anesthesia on the assessment of the HF model.

**Figure 2:**
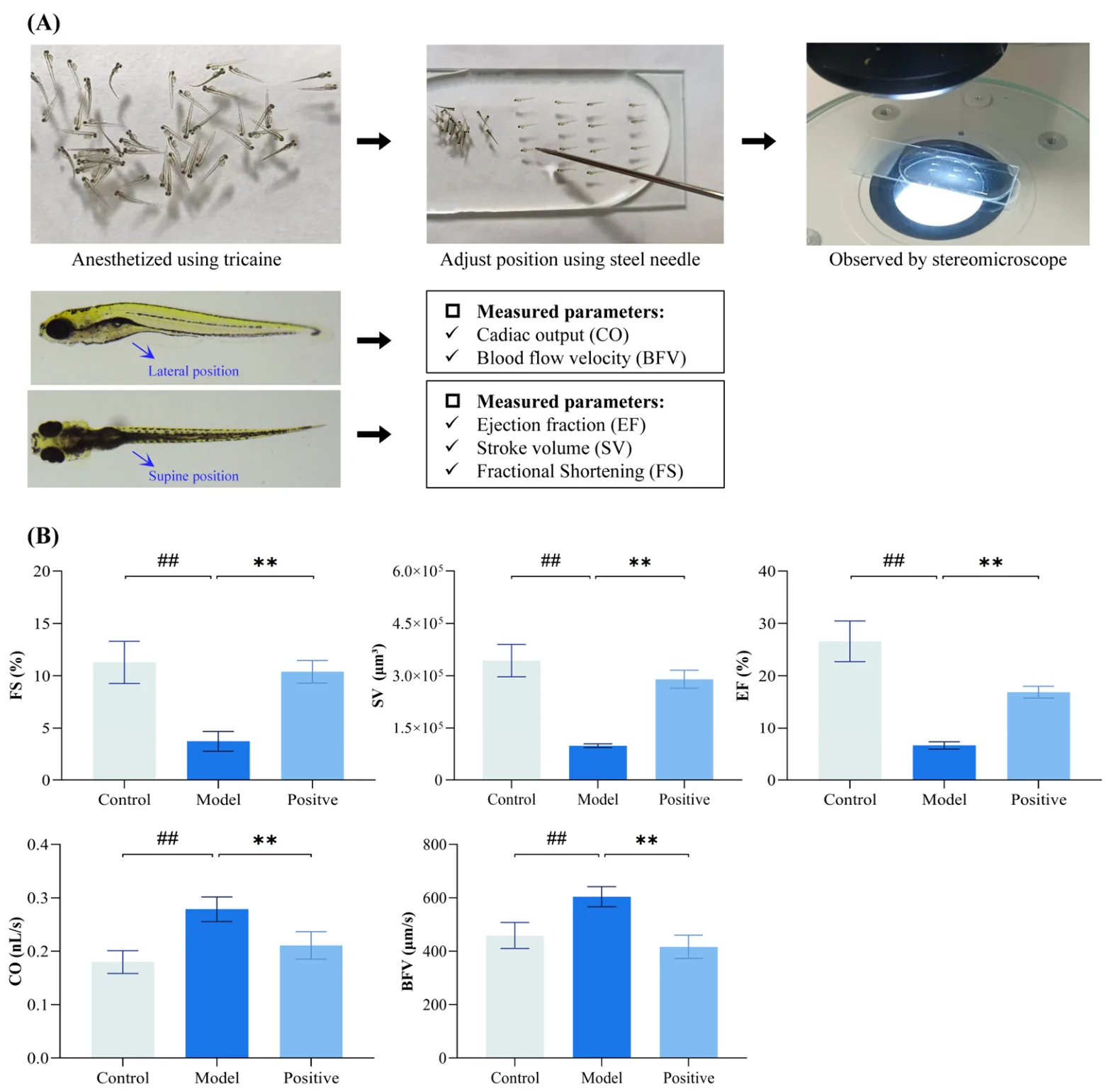
Traditional MS-222 anesthesia-based immobilization workflow and corresponding cardiac parameter detection. (**A**) Operation procedures for larval fixation via tricaine anesthesia combined with methylcellulose embedding and fine needle positioning. (**B**) Quantitative statistical results of five cardiac indicators obtained under standard MS-222 anesthetic conditions in the control, HF model and positive control groups (see Table S1). ^##^ *p* < 0.01 vs. the control group; ** *p* < 0.01 vs. the model group.

Direct parameter comparison revealed that MS-222 anesthesia significantly underestimated the severity of ISO-induced cardiac dysfunction across all five parameters in the HF model, with underestimation ranging from 11.3% to 49.3% (Table 1; see Tables S1 and S2 for full values). In addition, qualitative comparison of the pathological phenotypes showed that anesthesia partially obscured ISO-induced ventricular wall motion abnormalities, and that the degree of ventricular dilation was less pronounced under anesthesia than under microfluidic photography (Figure 2).

**Figure 3:**
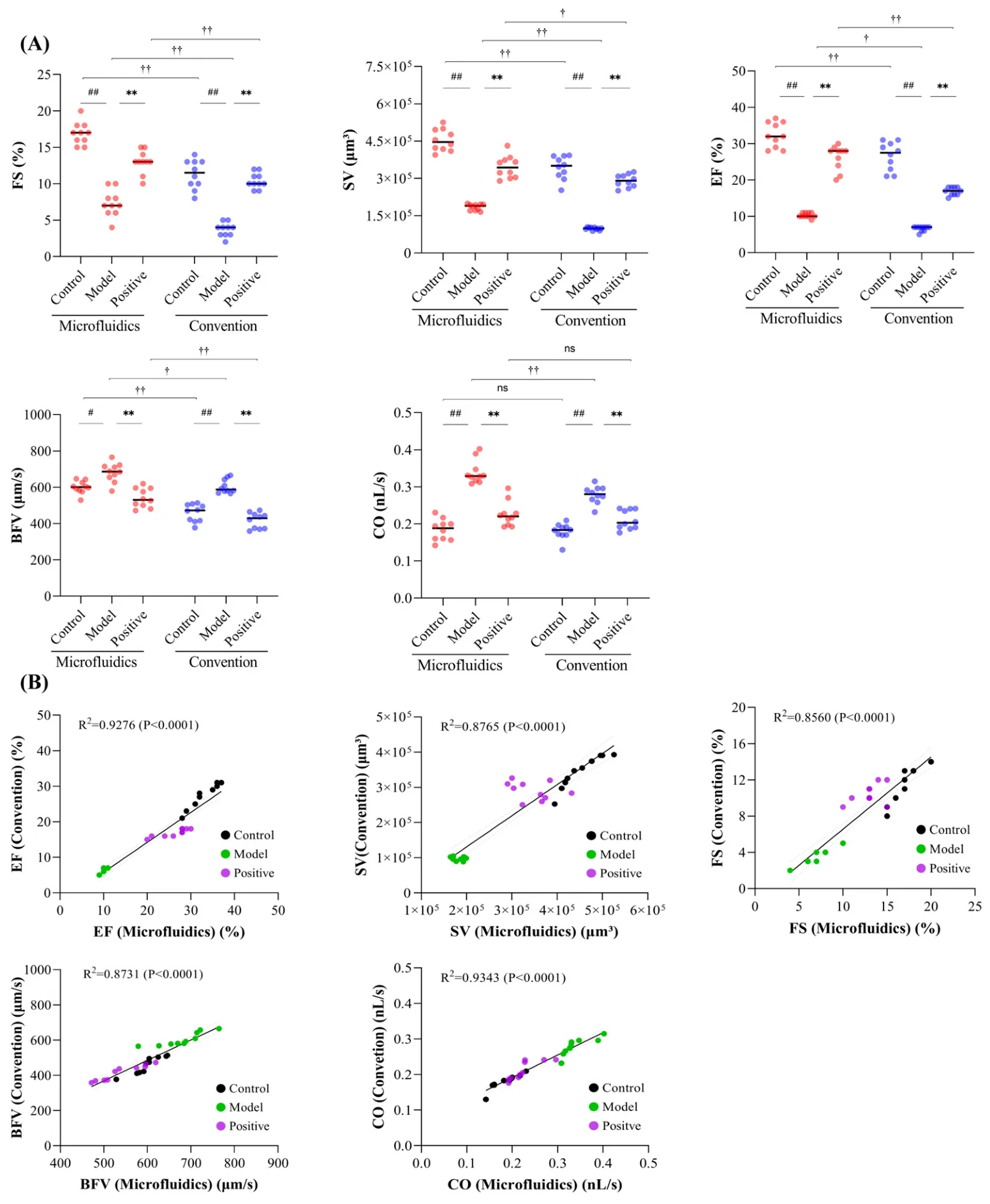
Dual factor intergroup analysis and correlation analysis. (**A**) Comparison of the five parameters between the control, HF, and propranolol-treated groups measured by the two methods. (**B**) Scatter plots and regression lines showing strong correlations between microfluidic and MS-222 anesthesia values for BFV and CO. All correlations were statistically significant (*p* < 0.0001). ** *p* < 0.01 indicate significant differences versus the control group; ^#^ *p* < 0.05 and ^##^ *p* < 0.01 indicate significant differences versus the HF model group; ns indicates no significant difference; ^†^ *p* < 0.05 and ^††^ *p* < 0.01 indicate significant differences between the two imaging methods (microfluidic vs. MS-222 anesthesia) within each treatment group.

Method agreement: The Pearson correlation between the two methods was strong for all the parameters (R^2^ > 0.85; *p* < 0.0001; Table 2), but the systematic underestimation confirmed that anesthesia introduces substantial bias in the assessment of cardiac function in ISO-induced HF models (Figure 3).

## 4. Discussion

### 4.1. Core Findings and Scientific Significance of This Study

In this study, we successfully developed a novel microfluidic platform for anesthesia-free, noninvasive in vivo photography of zebrafish HF models, addressing the long-standing critical limitation of anesthesia-induced cardiovascular artifacts in traditional photography approaches [18]. The platform achieved efficient immobilization of larval zebrafish via tapered microchannel hydrodynamic confinement, with an immobilization success rate of >95% and no procedure-related mortality observed during the 1 h experimental period [23]. Compared with the traditional MS-222 anesthesia method, our platform resulted in a significantly greater EF (10.3 ± 0.7% vs. 6.6 ± 0.7%, *p* < 0.001) and BFV (681 ± 53 μm/s vs. 604 ± 38 μm/s, *p* < 0.001) and enabled clear visualization of HF-specific pathological phenotypes, including ventricular dilation and reduced wall thickening [15,36]. These results demonstrate that anesthesia-free photography more closely reflects physiological cardiac function than conventional anesthesia-based imaging. Consistent with this, previous studies have shown that comparable hydrodynamic trapping does not induce acute stress responses or compromise survival and development in larval zebrafish [34,37]. Notably, Tang et al. [37] directly compared microfluidic hydrodynamic immobilization with freely swimming controls and conventional manual embedding, reporting survival comparable to the free-swimming control and superior to manual embedding, with no significant increase in morphological abnormalities. Together, these findings indicate that the platform substantially improves the accuracy and reproducibility of cardiac function assessment. This technology provides a more reliable experimental platform for studying HF pathogenesis and evaluating cardioprotective agents.

### 4.2. Comparison with Existing Technologies and Innovation Analysis

Our findings are consistent with those of previous reports documenting the dose-dependent cardiovascular depressant effects of MS-222, including bradycardia and reduced myocardial contractility [18,19]. Compared with existing microfluidic zebrafish photography platforms [16,38], our study presents three distinct advantages. First, the platform was specifically optimized for the assessment of cardiac function in an HF model, allowing stable immobilization without restriction of cardiac contraction. Second, it was fabricated using standard soft lithography techniques, featuring simple operation and low cost, facilitating widespread adoption in general laboratories. Third, the platform supports a photography throughput of approximately 60 larvae per hour, meeting the basic requirements for preliminary drug screening. Notably, unlike mechanical immobilization methods such as agarose embedding, hydrodynamic confinement causes no physical compression or stress responses in zebrafish, further enhancing the physiological relevance of the experimental results [34,39].

### 4.3. Study Limitations and Future Perspectives

This study has several limitations that warrant further investigation. First, the current platform is only suitable for zebrafish larvae at 3–7 dpf, and its performance for larger juvenile or adult fish requires optimization [14]. Second, the current throughput of approximately 60 larvae per hour remains insufficient for ultrahigh-throughput drug screening applications. Third, we validated the platform only in drug-induced HF models (ISO), and its applicability to genetic HF models or other cardiovascular disease models needs further verification [6]. Fourth, although previous studies have shown that comparable hydrodynamic trapping does not induce acute stress responses or compromise survival and development in larval zebrafish [34,37], we did not directly quantify stress markers (e.g., cortisol levels or immediate post-trapping heart rate spikes) in this study; the direct quantification of such stress indicators represents a limitation to be addressed in future work. Future work will focus on improving platform throughput and compatibility. Currently, manual loading of larvae into the chip yields a throughput of approximately 60 larvae per hour; to scale this platform toward high-throughput screening, we will focus on integrating automated fluidic switching systems, which would enable continuous, sequential loading and unloading of larvae without manual intervention. In addition, parallelizing the chip into multi-channel arrays and coupling the platform with automated stage control and robotic liquid handling would further increase throughput to meet the demands of large-scale pharmacological screening. Integrating these advances with high-content photography systems and artificial intelligence analysis technologies will support the development of a fully automated zebrafish HF drug screening platform. Additionally, this technology can be extended to other research areas requiring in vivo photography, such as developmental biology and toxicology, demonstrating broad application prospects [5,6].

## 5. Conclusions

In this study, a microfluidic platform was developed to facilitate dual-position (lateral and supine) in vivo photography of zebrafish heart failure models without the use of anesthesia. This platform enables the precise and concurrent measurement of five critical cardiac functional parameters—CO, BFV, EF, SV, and FS—thereby avoiding anesthesia-associated artifacts. These findings indicate that anesthesia significantly underestimates the severity of ISO-induced cardiac dysfunction, whereas the microfluidic approach yields data that more closely reflect physiological cardiac function. This technology may serve as a reliable tool for improving the accuracy of heart failure phenotypic characterization and screening for cardiovascular drugs.

## 6. Patents

The method in this paper has been filed as a Chinese invention patent (Application No. 202611078223.9, filed 20 July 2026) and accepted by the China National Intellectual Property Administration.

## Figures and Tables

**Table 1:** Comparison of cardiac function parameters between the microfluidic and anesthesia methods in ISO-induced HF larvae.

Parameter (Unit)	Microfluidic (Anesthesia-Free)	MS-222 Anesthesia	Difference (%) *
CO (nL/s)	0.339 ± 0.032	0.279 ± 0.023	17.7%
BFV (μm/s)	681 ± 53	604 ± 38	11.3%
EF (%)	10.3 ± 0.7	6.6 ± 0.7	35.9%
SV (μm^3^)	184,519 ± 12,822	97,979 ± 5306	46.9%
FS (%)	7.3 ± 1.8	3.7 ± 0.9	49.3%

* Difference (%) = (Microfluidic anesthesia-free value − MS-222 anesthesia value)/Microfluidic anesthesia-free value × 100%, representing the percentage of parameter underestimation resulting from MS-222 anesthesia.

**Table 2:** Correlation analysis of parameters between the microfluidic and anesthesia methods.

Parameter (Unit)	Pearson R^2^	Regression Equation (Y = Anesthesia, X = Microfluidic)
BFV (μm/s)	0.8731	Y = 1.157 × X − 209.5
CO (nL/s)	0.9343	Y = 0.6304 × X + 0.06573
EF (%)	0.9276	Y = 0.8422 × X − 2.642
SV (μm^3^)	0.8765	Y = 0.8841 × X – 45,930
FS (%)	0.8560	Y = 0.7964 × X − 1.408

## Data Availability

The raw data supporting the conclusions of this article will be made available by the authors upon request.

## Supplementary Materials

The following supporting information can be downloaded at https://www.mdpi.com/article/10.3390/biomedicines14092005/s1, Table S1: Cardiac function parameters measured under MS-222 anesthesia; Table S2: Cardiac function parameters measured by the microfluidic platform (anesthesia-free).

## Abbreviations

The following abbreviations are used in this manuscript:

HF	Heart failure
ISO	Isoproterenol
MS-222	Tricaine methanesulfonate
CVDs	Cardiovascular diseases
dpf	Day post-fertilization
PDMS	Polydimethylsiloxane
CO	Cardiac output
BFV	Blood flow velocity
EF	Ejection fraction
SV	Stroke volume
FS	Fractional shortening
DA	Dorsal aorta
EDV	End-diastolic volume
ESV	End-systolic volume
EDD	End-diastolic diameter
ESD	End-systolic diameter
