# Supplementary material for: An Anesthesia-Free Pharmacological Assay for Zebrafish Heart Failure Models Using a Microfluidic Platform
Source: Biomedicines. 2026 Sep 6;14(9):2005. doi: 10.3390/biomedicines14092005 (PMC13604212; doi:10.3390/biomedicines14092005)
Supplement: Supplementary file 1 [file biomedicines-14-02005-s001.zip › biomedicines-4509037-supplementary.pdf]

Supplementary Materials

An Anesthesia-Free Pharmacological Assay for Zebrafish Heart Failure Models Using a Microfluidic Platform

Qiuyue Song [1†], Fangrui Liu [2†], Jinyan Zhao [1], Wenli Xie [1], Xianjun Fu [1,2,*] and Kuo Xu [1,2,3,*]

1 Research Institute of Marine Traditional Chinese Medicine (Qingdao Academy of Chinese Medical Sciences), The SATCM's Key Unit of Discovering and Developing New Marine TCM Drugs, Key Laboratory of Marine Traditional Chinese Medicine in Shandong Universities, Shandong University of Traditional Chinese Medicine, Qingdao 266114, China

2 College of Pharmacy, Shandong University of Traditional Chinese Medicine, Jinan 250355, China

3 National Center of Technology Innovation for Comprehensive Utilization of Saline-Alkali Land, Dongying 257345, China

* Correspondence: xianxiu@hotmail.com (X.F.); xukuoworld@126.com or xukuoworld@sdutcm.edu.cn (K.X.)

† These authors contributed equally to this work.

Table S1. Cardiac function parameters measured under MS-222 anesthesia.

Parameter (unit)	Control	ISO (Model)	Propranolol (Positive)
CO (nL/s)	0.180 ± 0.021	0.279 ± 0.023	0.211 ± 0.026
BFV (μm/s)	459 ± 49	604 ± 38	416 ± 44
EF (%)	26.6 ± 3.9	6.6 ± 0.7	16.9 ± 1.1
SV (μm^3)	341,769 ± 46,657	97,979 ± 5,306	290,455 ± 25,822
FS (%)	11.3 ± 2.0	3.7 ± 0.9	10.4 ± 1.1

Values are expressed as mean ± SD (n = 10 larvae per group, three independent experiments). One-way ANOVA followed by Tukey's post-hoc test was used for comparisons among Control, ISO model and Propranolol groups under MS-222 anesthesia (GraphPad Prism version 9.5; San Diego, CA, USA). Significant differences ($p < 0.05$) are marked in bold in main figures.

Table S2. Cardiac function parameters measured by the microfluidic platform (anesthesia-free).

Parameter (unit)	Control	ISO (Model)	Propranolol (Positive)
CO (nL/s)	0.184 ± 0.088	0.339 ± 0.032	0.226 ± 0.034
BFV (μm/s)	600 ± 35	681 ± 53	541 ± 52
EF (%)	32.4 ± 3.4	10.3 ± 0.7	26.2 ± 3.4
SV (μm^3)	453,511 ± 44,041	184,519 ± 12,822	345,982 ± 45,081
FS (%)	16.9 ± 1.5	7.3 ± 1.8	13.0 ± 1.6

Values are expressed as mean ± SD (n = 10 larvae per group, three independent experiments). One-way ANOVA followed by Tukey's post-hoc test was used for comparisons among Control, ISO model and Propranolol groups detected by the anesthesia-free microfluidic platform (GraphPad Prism version 9.5; San Diego, CA, USA). Significant differences ($p < 0.05$) are marked in bold in main figures.
